# Towards controlled polymer brushes via a self-assembly-assisted-grafting-to approach

**DOI:** 10.1038/ncomms11119

**Published:** 2016-03-24

**Authors:** Tian Zhou, Hao Qi, Lin Han, Dmitri Barbash, Christopher Y. Li

**Affiliations:** 1 Department of Materials Science and Engineering, Drexel University, Philadelphia, Pennsylvania 19104, USA; 2 School of Biomedical Engineering, Science and Health Systems, Drexel University, Philadelphia, Pennsylvania 19104, USA

## Abstract

Precise synthesis of polymer brushes to modify the surface of nanoparticles and nanodevices for targeted applications has been one of the major focuses in the community for decades. Here we report a self-assembly-assisted-grafting-to approach to synthesize polymer brushes on flat substrates. In this method, polymers are pre-assembled into two-dimensional polymer single crystals (PSCs) with functional groups on the surface. Chemically coupling the PSCs onto solid substrates leads to the formation of polymer brushes. Exquisite control of the chain folding in PSCs allows us to obtain polymer brushes with well-defined grafting density, tethering points and brush conformation. Extremely high grafting density (2.12 chains per nm^2^) has been achieved in the synthesized single-tethered polymer brushes. Moreover, polymer loop brushes have been successfully obtained using oddly folded PSCs from telechelic chains. Our approach combines some of the important advantages of conventional ‘grafting-to' and ‘grafting-from' methods, and is promising for tailored synthesis of polymer brushes.

## Abstract (web summary)

Polymer brushes, whereby single layers of polymers are tethered to surfaces, can be made by various methods but greater control over their fabrication is desirable. Here, by pre-assembling polymer chains into 2D crystalline sheets, the authors achieve high precision and unprecedented grafting densities.

## Graphical abstract

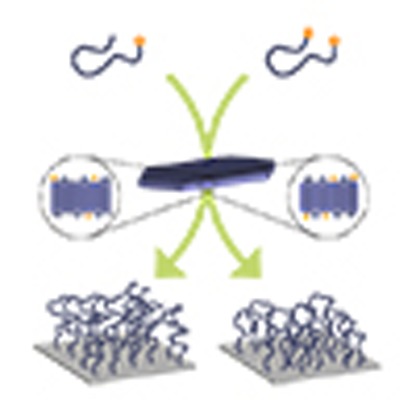

Long-chain polymer molecules can be attached at their chain end(s) to a surface or interface by either physical or chemical bonds. Depending on the surface grafting density of these polymer chains, their morphology can be described as brushes, mushrooms or pancakes^1^,^2^. In polymer brushes, because the distance between the neighbouring tethering points is small, there is significant overlay among adjacent polymer chains, and pronounced chain stretching takes place orthogonal to the substrate. The past few decades witnessed exciting progresses in studies on polymer brushes, and they show great promises in various fields, including coating, biomedical, sensing, catalysis and so on^3^,^4^,^5^,^6^,^7^,^8^,^9^. On the synthesis side, two major synthetic approaches, including grafting-to and grafting-from, have been developed, with clear advantages and disadvantages associated with each of the method^10^,^11^,^12^,^13^. For example, a grafting-from approach produces dense polymer brushes with large thickness, but the chemical reactions required are usually complex and the as-prepared polymer brushes need to be detached from the substrate for characterization purposes^14^,^15^,^16^,^17^,^18^. Using grafting-to approaches, the chemical structures and dispersity (*Ð*) of polymers can be better controlled/characterized. However, the resulting grafting density is strongly dependent on the molar mass of the polymer chains; polymer brushes with high grafting density are challenging to achieve using this method. In this paper, we disclose an approach, which is fundamentally different from the previously reported methods, to synthesize polymer brushes with controlled structure and high grafting density on flat surfaces. In this approach, we first pre-assemble end-functionalized polymers into two-dimensional (2D) polymer single crystals (PSCs) using controlled crystallization. These 2D single-crystal sheets can then be covalently coupled onto a flat substrate. On removing unbound polymers, well-defined polymer brushes can be obtained. We therefore name the approach ‘self-assembly-assisted-grafting-to' method.

PSCs have been extensively investigated during the past 60 years. They are typically used as the model system to determine polymer crystalline structures and related polymer physics^19^,^20^,^21^,^22^. Recently, we demonstrated that surface-functionalized 2D PSCs can be considered as free-standing ‘self-assembled monolayers'—they can collect nanoparticles, and the resultant hybrid materials can be used in applications such as nanoparticle asymmetric functionalization, nanomotors, surface-enhanced Raman spectroscopy and catalysis supports^23^,^24^,^25^,^26^,^27^,^28^. These surface-functionalized 2D single crystals also provide a unique opportunity for the synthesis of well-defined polymer brushes. The key step in our method is pre-assembling polymers into PSCs before coupling them onto the substrate. More importantly, these PSCs should adapt a so-called integral folding conformation, that is, the number of folds for each polymer chain should be an integral number (*n*)^21^. This unique conformation ensures the end-functional groups reside on the surface of the crystal for subsequent chemical coupling. As the first demonstration of this method, alkoxysilane-terminated poly (ɛ-caprolactone) (PCL) was used as the model system to grow the PSC templates whose thickness was tuned by the crystallization temperature (*T*_c_). PCL single-crystal templates with different thicknesses were covalently bound to a glass substrate, yielding polymer brushes with well-controlled grafting density and tethering points. Exceptionally high grafting density (∼2.12 chains per nm^2^) has been achieved by using extended chain single crystals as the templates. Furthermore, using bifunctional telechelic PCL to form oddly folded PSCs, we were able to synthesize polymer loop brushes (PLBs) with precisely controlled tethering points and high grafting density (0.53 chain per nm^2^ and 1.06 tethering points per nm^2^). Compared with previously reported conventional brush synthesis approaches, our strategy combines the advantages of both grafting-to and grafting-from methods, leading to polymer brushes with well-controlled structures and high grafting density.

## Results

### Polymer brushes with precisely tuned grafting density

**Figure 1:**
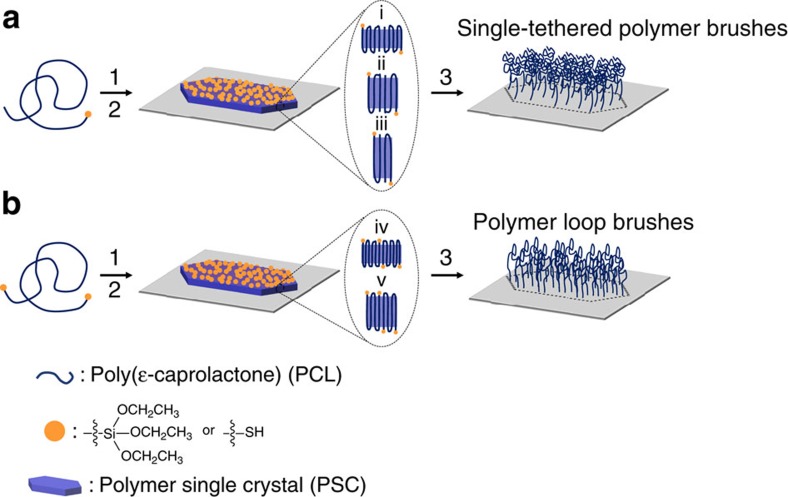
Synthetic route for polymer brushes via the self-assemble-assisted-grafting-to method. (**a**) Synthesis of STPBs using mono-functionalized PCL; (**b**) preparation of PLBs using α, ω-functionalized PCL. Steps 1, 2 and 3 are solution crystallization via a self-seeding method, chemical immobilization of PSCs on a solid substrate and the removal of unbound polymer chains, respectively. The integral number folding structure of polymer chains is illustrated in (i–v), and *n*=4, 2, 1, 5 and 3 for i–v, respectively. In the process of **b**, oddly folded chain structure is required for synthesizing PLBs, as shown in (iv) and (v). The dotted line on the substrates represents the outline of polymer brush-grafted regime templated from the hexagonal-shaped PSC, indicating chemical tethering of polymer brushes only takes place at the interface between PSCs and the solid substrate.

**Figure 2:**
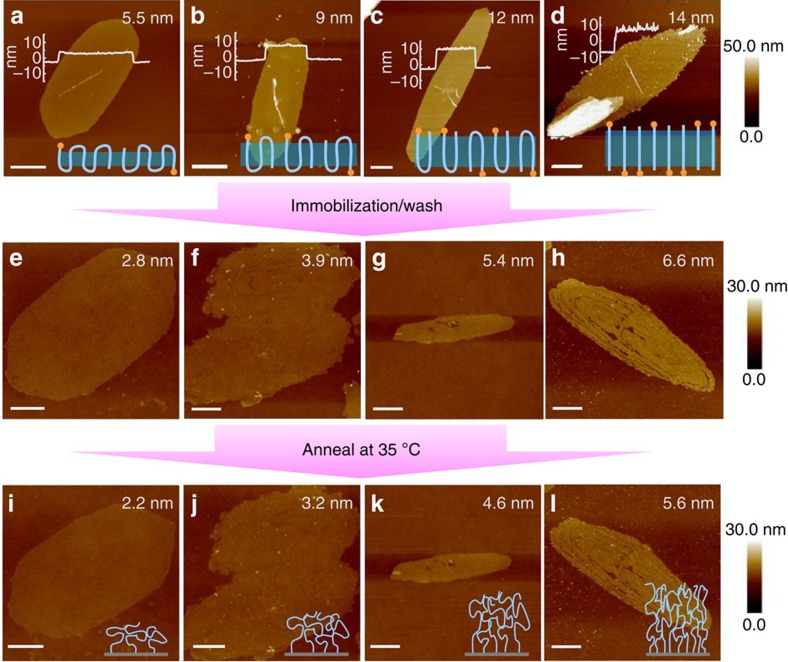
Precise tuning of brush-grafting densities. Tapping-mode-AFM height images of TEG-PCL30-SiOR single crystals grown in dilute 1-butanol solution at 5 (**a**), 15 (**b**) and 35 °C (**c**), and TEG-PCL16-SiOR single crystals grown at 30 °C (**d**). The inset curves show the height profile of the corresponding crystal lamellae, and the schematics illustrate the chain-folding behaviours. Folding numbers are 4, 2, 1 and 0 for **a**–**d**, respectively. (**e**–**g**,**i**–**k**) The corresponding PCL brushes prepared from TEG-PCL30-SiOR single-crystal templates before (**e**–**g**) and after (**i**–**k**) thermal annealing. (**h**,**l**) PCL brushes prepared from TEG-PCL16-SiOR single-crystal templates before (**h**) and after (**l**) annealing. Scale bars, 1 μm. The inset numbers on the top right corner of each image are the measured thicknesses using AFM.

The synthetic route of polymer brushes via self-assembly-assisted-grafting-to method is shown in Fig. 1. In this study, alkoxysilane group was used to functionalize the chain ends to form chemically tethered polymer brushes on glass substrates. Hydroxyl-terminated PCL (PCL-OH) was first synthesized via ring-opening polymerization using tetraethylene glycol monomethyl ether as the initiator followed by chain end functionalization using 3-(triethoxysilyl)propyl isocyanate. Supplementary Fig. 2 summarizes the proton NMR (nuclear magnetic resonance) and gel permeation chromatography (GPC) results of the functionalized PCL. According to the chain end analysis using NMR, the degree of polymerization of the polymer is ∼30 (denoted as TEG-PCL30-SiOR), while GPC shows the *Ð* is 1.22. Solution crystallization via self-seeding was then used to prepare PSCs following the temperature programme as shown in Supplementary Fig. 1 and Supplementary Table 1. The seeding temperature (*T*_s_) is critical to forming uniform single crystals, and in this work *T*_s_ was fixed at 42 °C, while three different *T*_c_ were chosen to prepare PSCs with different lamellar thicknesses. Figure 1 shows the atomic force microscopy (AFM) images of the resultant TEG-PCL30-SiOR single crystals at *T*_c_=5, 15 and 35 °C, respectively. In all of the three cases, hexagonal-shaped single crystals have been obtained. The shape slightly varies and the aspect ratio of the crystal changes from 2/1 to 3/1 and 5/1 as the *T*_c_ increases from 5 to 15 and 35 °C. The increased aspect ratio is due to the change of the relative growth rates of each crystal planes as the *T*_c_ increases^19^,^20^,^21^. More interestingly, height analysis of the AFM images shows the lamellar thickness of these PSCs is 5.5, 9 and 12 nm, respectively, which is due to the different undercooling during the crystallization process^29^. PCL has an orthorhombic unit cell, with *a*=0.747 nm, *b*=0.498 nm and *c*=1.705 nm, space group of P2_1_2_1_2_1_ and a molecular helix of 7*2_1_ (ref. 30). Calculation based on this unit cell structure of PCL and the degree of polymerization suggests that the polymer chains fold 4, 2 and 1 times for the three crystals, respectively (schematics at the bottom of Fig. 2a–c). Since all the polymer chains adopt integral folding number, their alkoxysilane chain ends should be exclusively exposed on the lamellar surfaces, and can then serve as reaction sites for the following immobilization reaction. On the basis of the chain-folding structure of each crystal lamella, the areal density of alkoxysilane on one lamellar surface can be calculated to be 0.53, 0.89 and 1.33 -SiOR per nm^2^, respectively. If we assume all the alkoxysilane groups on one side of the lamellae can be chemically coupled onto a glass substrate, these numbers are then the theoretical grafting density of the as-prepared polymer brushes.

The above-mentioned PCL single crystals were spin-coated onto a glass substrate. Ammonia was then applied to catalyse the solid-state reaction between silanol groups on the glass surface and the exposed alkoxysilane groups on the crystal lamellae. After 20 min of reaction, polymer-coated slides were thoroughly washed with acetone to remove unbound polymer chains. The as-prepared polymer brushes were then characterized using AFM as shown in Fig. 2e–g. It can be noticed that the polymer brush-covered domain retains the original hexagonal shape of the PSCs. Height analysis of the as-prepared polymer brush domains indicates the thickness significantly decreases compared with the corresponding single-crystal lamellae. This is because during solution crystallization, polymer chain ends are randomly exposed on the two surfaces of the 2D PSC (schematics in Fig. 2a–c). If all the alkoxysilane chain ends on one side of the lamella were successfully coupled with the substrate, we then anticipate a decrease of the coating thickness to 50% of the original value. The experimental results show a slightly greater decrease (Fig. 2), which can be attributed to the unreacted chain ends during immobilization—when PSCs were deposited onto a substrate, because of the surface roughness of the PSCs, not all the alkoxysilane chain ends have intimate contact with the substrate to ensure a successful coupling.

**Figure 3:**
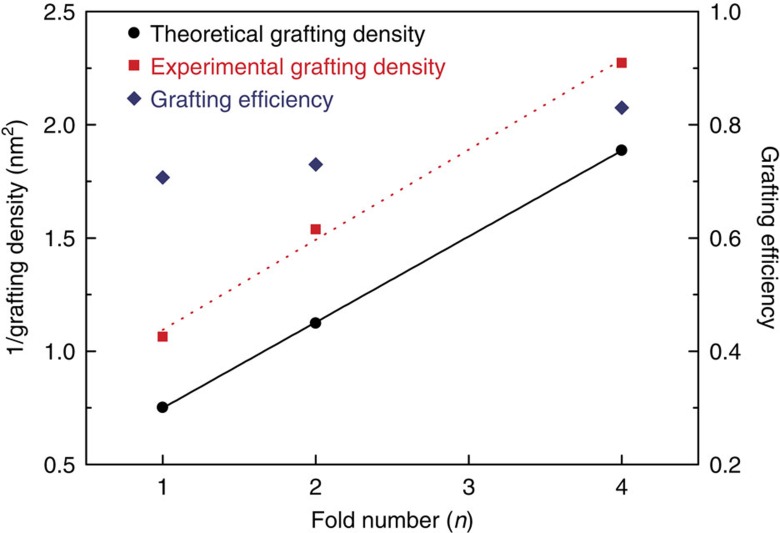
Chain-folding determines grafting density. Correlation between the grafting density of the polymer brushes and the folding number of the polymer single-crystal templates.

Grafting density of the PCL brush samples prepared using different PSC templates can be calculated using the following equation:


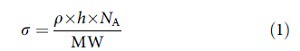


where *ρ* is the polymer density, *σ* is the grafting density, *h* is the polymer brush layer height, MW is the molar mass of polymer brushes and *N*_A_ is the Avogadro's number. Because the thickness measured using as-prepared PCL brushes can be inaccurate due to the possible ‘constrained dewetting', which could result in inhomogeneous coating during the evaporation of acetone after the washing process^31^,^32^, thermal annealing of the as-prepared polymer brush samples was performed overnight at 35 °C. This is the onset temperature of TEG-PCL30-SiOR crystallization according to the differential scanning calorimetry (DSC) thermogram (Supplementary Fig. 2c), annealing at this temperature leads to the crystallization of the PCL brushes, which eventually removes excessive free volume in the polymer brush coating, hence more precise brush density calculation. Height images obtained from AFM of these annealed samples are summarized in Fig. 2i–k. Compared with as-prepared polymer brushes, morphology of the polymer brush retained, while the thickness of annealed polymer brushes slightly decreased with a smoother surface according to image analysis results summarized in Table 1. Calculation of polymer brush grafting density was then performed based on the annealed sample thickness, which yields the grafting densities of 0.44, 0.65 and 0.94 chain per nm^2^. Figure 3 shows the linear correlation of the grafting density with the chain-folding number *n* in the PSCs. The results therefore demonstrate the capability of precise control over the grafting density of the polymer brushes formed by a given polymer using our method. Note that the polymer chain grafting density calculated from AFM characterizations is slightly smaller than the grafting density of -SiOR groups on the single crystals, which is likely due to the reaction efficiency issue during solid-state coupling between single-crystal lamellae and glass substrates. We can define the coupling efficiency (*f*) as the ratio between the experimental and theoretical grafting densities. For PSCs with three thicknesses, *f* can be found to be 0.71, 0.73 and 0.78, respectively.

Polymer brushes with a grafting density >1 chain per nm^2^ represent extremely dense brushes, and have been rarely reported even using the grafting-from approach^33^,^34^,^35^,^36^. The highest grafting density achieved in the above-mentioned system is 0.94 chain per nm^2^. Figure 3 suggest that if extended-chain single crystals (*n*=0) prepared from mono-functionalized PCL are used as templates, the grafting density can be further increased. To test this hypothesis, single crystals from PCL with degree of polymerization of 16 (TEG-PCL16-SiOR, see Supplementary Fig. 2 for characterizations of its chemical structure) was prepared via the self-seeding method at *T*_c_=30 °C. As shown in Fig. 2d, the obtained lamellar thickness is ∼14 nm, which confirms that extended-chain structure has been achieved. In this case, the surface areal density of alkoxysilane groups on one side of the lamellar surface is 2.67 –SiOR per nm^2^, which in theory is the highest for symmetric mono-functionalized PCL crystals. Using the same ammonia-catalysed solid-state coupling reaction, the polymer brushes were successfully prepared and studied using AFM. As seen in Fig. 2h, the as-prepared brush domain has a thickness of ∼6.6 nm, which decreased to 5.6 nm after thermal annealing at 35 °C (Fig. 2l). The grafting density of the polymer brushes can therefore be estimated to be ∼2.12 chains per nm^2^ using equation (1). Compared with other studies on the synthesis of polymer brushes on planar surfaces using either grafting-to or grafting-from methods, the grafting density of polymer brushes prepared using our method can reach a considerably higher value.

**Figure 4:**
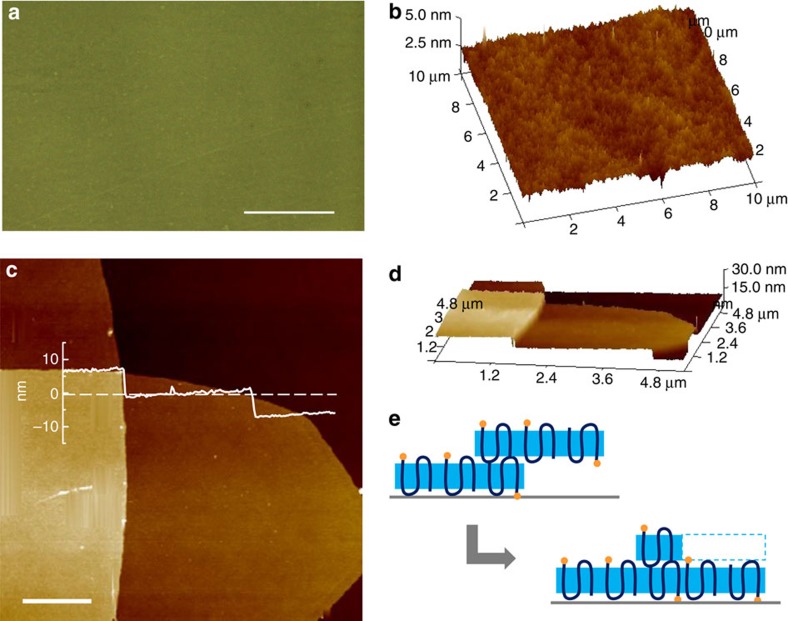
Uniform coating of the polymer brushes at a macroscopic length scale. (**a**) Phase-contrast optical microscopy image of polymer brush-coated glass surface prepared using TEG-PCL30-SiOR single crystal grown at 15 °C as templates. Large surface grafting can be achieved with small amount of unmodified area showing relatively lighter colour compared with brush-covered regime; (**b**) three-dimensional (3D) height images of a 10 × 10-μm AFM scan randomly picked from image **a**; (**c**) AFM height image shows the stacking of two TEG-PCL30-SiOR PSCs prepared at 15 °C. The inset of **c** shows the height profile of the crystal lamellae, and the sudden change (instead of gradual decrease) in height at the edge of overlapped area indicates chain sliding. (**d**) 3D height image of **c**; (**e**) schematic illustration of the chain sliding when two PSC lamellae stack with each other when deposited onto a solid substrate. Scale bars, 50 μm (**a**); 1 μm (**c**).

PSCs can be conveniently deposited onto a glass substrate to prepare a uniform, large-scale coated surface. As an example, TEG-PCL30-SiOR PSCs grown at 15 °C were drop casted onto a 5 mm × 5 mm glass slides. After the residual solvent was evaporated, similar solid-state coupling reaction was conducted to form chemically bounded polymer brushes on the glass slide. Over 10 random areas from the substrate were subject to phase-contrast optical microscopy imaging, and a typical graph is shown in Fig. 4a. Nearly 100% of the surface is covered with polymer brushes within this large area (scale bar, 50 μm). Furthermore, AFM characterizations were also applied to study the nanostructures of polymer brushes prepared herein as shown in Fig. 4b. The polymer brush-covered area is homogeneous with a surface roughness comparable to those prepared using isolated PSCs as templates summarized in Table 1. Phase-contrast microscopy and AFM experiments demonstrate that our method can be used to functionalize a macroscale surface with a uniform coverage. Of interest is that no obvious ‘grain boundaries' between PSCs were observed in AFM images. When casting PSCs onto a solid substrate, one expects that due to the defined shape/sizes, regions between adjacent PSCs might not be coated with brushes as shown in Fig. 4c. The observed uniform coating of the PSCs can be explained by the combination of elasto-capillary bending and chain sliding within PSCs^19^,^20^,^21^,^37^,^38^. For overlapped PSCs as shown in Fig. 4c, on solvent evaporation, the elasto-capillary bending would cause the top PSC deform towards the substrate, and the resultant interface width can be estimated as ∼190 nm (ref. 39). However, detailed AFM analysis in Supplementary Fig. 4 suggests that the collapsed interface has an average width of ∼33 nm (average of 10 measurements). The further sharpening of the interface can be explained by the chain sliding in the PSC caused by the capillary force. Because of the weak van der Waals force between adjacent chains in the PSC, it is relatively easy for chains to slide among each other^40^. As shown in Fig. 4d,e, as stacking occurs, the polymer chains of the top lamella slide down and fill in the bottom areas. This chain sliding apparently accounts for the filling of the otherwise ‘empty' surface due to lamella stacking; it leads to the formation of uniform and dense polymer brushes.

### Synthesis of dense PLBs

**Figure 5:**
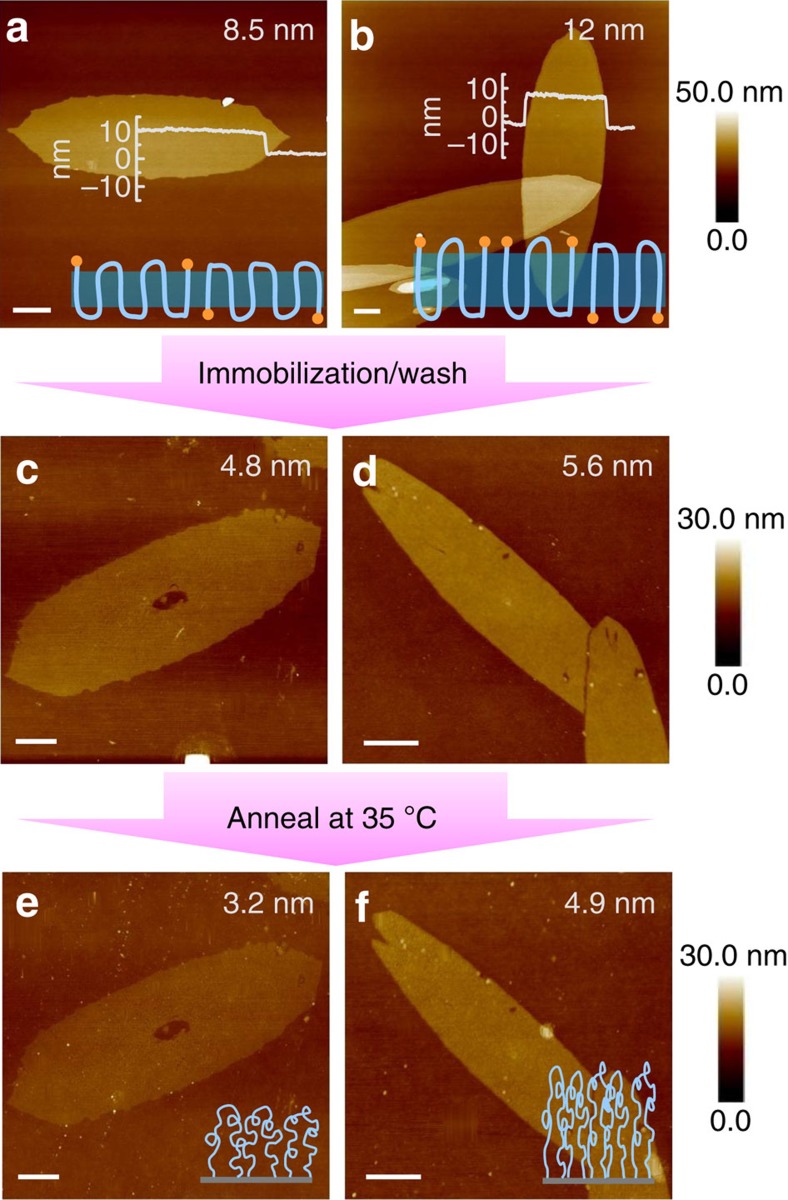
Controlled grafting of dense polymer loops. Tapping-mode-AFM height images of polymer single crystals prepared from diPCL-2SiOR at 5 (**a**) and 35 °C (**b**). The inset curves show the corresponding height profile of crystal lamellae, and the schematics illustrate the oddly folded chain conformations (*n*=3 and 5). (**c**–**f**) The corresponding PLBs before (**c**,**d**) and after (**e**,**f**) annealing. The schematics illustrate the polymer loops with different grafting density. Scale bars, 1 μm. The inset numbers on the top right corner of each image are the measured thicknesses using AFM.

Another important polymer conformation on substrate is PLBs, where the polymer chain is tethered via both chain ends. The chain conformation, surface dynamics, friction and mechanical response of PLBs are found quite different compared with their single-tethered polymer brush (STPB) counterparts^41^,^42^,^43^,^44^,^45^,^46^,^47^,^48^. Detailed experimental studies on these materials however have been quite limited due to the lack of efficient approach towards uniform PLBs coating with precisely controlled ring size, molar mass and high grafting density. Up till now, only solution-based physisorption of triblock terpolymer^49^,^50^,^51^,^52^ and grafting-to of telechelic polymers^53^,^54^,^55^,^56^ have been successfully applied for the synthesis of PLBs on flat surfaces. Yet it is challenging to precisely control final brush structures and grafting density. Reversible addition-fragmentation chain transfer polymerization has been used to synthesize poly(methyl acrylate) loops on silica nanoparticle; chain branching of the brushes was noticed due to the close proximity of growing radicals^57^. Our method can also be used to synthesize PLBs with well-controlled anchoring points and high grafting density. To this end, α, ω-alkoxysilane-terminated telechelic PCL (diPCL-2SiOR) was used for PSC preparation. The chemical structures of these polymers were systematically studied using NMR and GPC as shown in Supplementary Fig. 3, which confirmed the average degree of polymerization of these PCL was 56, while the Ð was ∼1.3. When a telechelic polymer chain folds odd-number times in the crystal lamellae, both chain ends are exposed onto the same side of lamellar surfaces as illustrated in Fig. 1b. After depositing such PSCs onto a solid substrate, coupling chain ends to the solid substrate would lead to PLBs. In our experiment, the lamellar thickness of the single crystals was adjusted by changing the *T*_c_ as shown in Fig. 5a,b. When PCL was crystallized at 5 °C, the obtained lamellae thickness was 8.5 nm, which increased to 12 nm when crystallized at 35 °C. These two lamellar thicknesses correspond to five and three times chain folding, respectively. Oddly folded chain structures were then successfully achieved. Using the similar solid-state grafting-to method, polymer brushes were successfully obtained. Figure 5 summarizes the AFM height images of the as-prepared polymer brushes templated from PSCs grown at 5 and 35 °C. Similar with the STPBs templated from PCL-SiOR PSCs, polymer brush domain herein also retained the same shape of crystal templates, and the as-prepared polymer brushes templated from diPCL-2SiOR PSCs prepared at 5 °C is about 4.8 nm in thickness, while the ones using PSCs grown at 35 °C show a dry thickness of around 5.6 nm.

**Figure 6:**
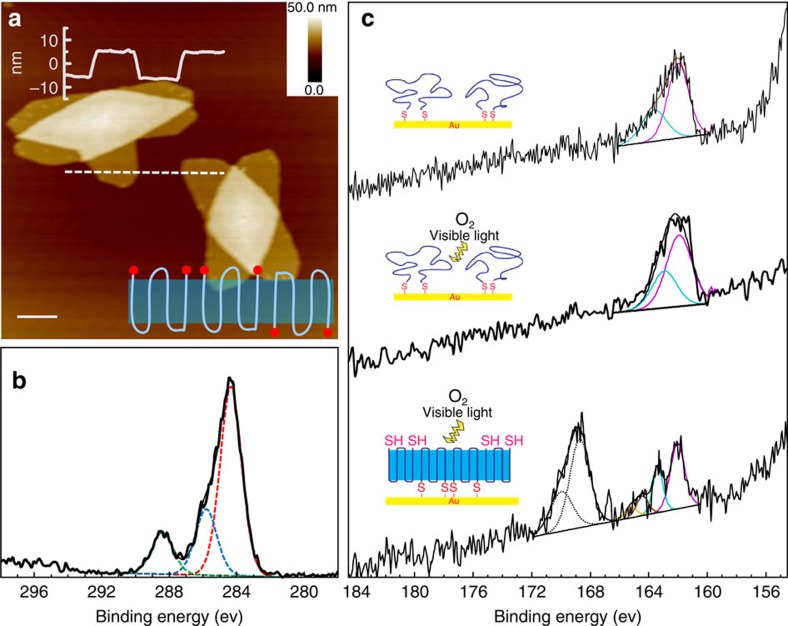
Confirmation of the polymer loop conformation. (**a**) AFM height image of single crystal grown from diPCL-2SH at 35 °C. The inset curve shows the height profile of crystal lamellae along the dotted line, and the cartoon illustrates the three-time chain-folding morphology of PCL molecule perpendicular to lamellae surface. Red solid spheres represent exposed thiol chain ends. Scale bar, 1 μm. (**b**) C1s HR-XPS spectrum and its deconvolution results of PCL brushes on Au substrate templated from diPCL-2SH PSCs crystallized at 35 °C; (**c**) S2*p* HR-XPS spectra and corresponding deconvolution results. From top to bottom: as-prepared PCL PLBs; PCL loop brushes after oxidation; and diPCL-2SH PSCs on Au substrate after oxidation. The insets illustrate the chemical structures of different sulfur species existed in polymer brushes and PSCs.

To further confirm polymer brushes prepared herein were indeed loops, a control experiment was designed using PSCs of dithiol functionalized PCL (diPCL-2SH, degree of polymerization ∼56 as calculated from NMR, see Supplementary Fig. 3) grown at 35 °C as the polymer brush templates. As seen in Fig. 6a, the thickness of crystal lamellae is the same as diPCL-2SiOR PSCs prepared at 35 °C, which confirms the identical chain-folding morphology within these two crystal lamellae (three-fold). In this case, polymer brushes prepared from these templates should have exactly the same morphology with those prepared from diPCL-2SiOR. Polymer brushes were then grafted onto a gold substrate using diPCL-2SH PSCs as templates via Au–S bonds, which formed readily under ambient condition followed by the removal of unbound polymer chains. The successful immobilization of PCL brushes was confirmed using high-resolution (HR) C1*s* X-ray photoelectron spectroscopy (XPS) spectrum as shown in Fig. 6b. The deconvolution of the signal clearly shows three different components with binding energies at about 284.6, 286.2 and 288.7 eV, attributed to the C–H, C–O and O=C–O species, respectively^58^. The area ratio of [C–H]:[C–O]:[O=C–O] is around 73:14:13, which is in good agreement with the theoretical value of 5:1:1 for the PCL structure^59^.

HR S2*p* XPS spectra were then employed to investigate the formation of chemical bonds between polymer chain ends and Au substrates to confirm whether these polymer brushes were singly or doubly tethered^54^. The chemical structures of the tethering sites of as-prepared polymer brushes were first examined as shown in the first spectrum of Fig. 6c with clear peaks of the 162/163.1 eV S2*p* spin–orbital pair (S2*p*, 3/2, 1/2), which are attributed to the Au–S bond formation, demonstrating successful chemical tethering of polymer brushes. Moreover, no free thiol-binding energy signals at 163.5/164.8 eV can be observed, which suggests all thiol groups at the polymer chain ends are chemically bounded, confirming the loop-structured brushes. This can be further supported by oxidizing the polymer brushes under ambient condition, and the spectrum after oxidation shown in the second spectrum of Fig. 6c does not exhibit any noticeable signals from oxidized thiol. A control sample prepared by skipping the washing process after immobilization of diPCL-2SH PSCs on Au substrate was subject to the same oxidation procedure, and the S2*p* HR-XPS spectrum of this sample is shown in the bottom spectrum of Fig. 6c. Other than similar thiolated sulfur signals, strong sulfonic acid peaks due to oxidized unbound thiol groups with binding energy of 169.1/170.3 eV (S2*p*, 3/2, 1/2) can be clearly seen in the curve. On the basis of these results, the PCL brushes prepared herein had all the chain ends being chemically tethered, hence forming loop conformation (PLBs).

The grafting density of PLBs templated from diPCL-2SiOR PSCs was also calculated based on the dry thickness obtained from AFM imaging using equation (1). Thermal annealing at 35 °C was conducted to remove free volume within brush layers, and the series of AFM images is summarized in Fig. 5. Similar effects on the thickness of polymer brushes and the surface roughness can be observed compared with STPBs described in the previous discussion. On the basis of the annealed layer thickness, the grafting density *σ* can be calculated as 0.35 and 0.53 loops per nm^2^ for PLBs templated from PSCs grown at 5 and 35 °C, respectively. Meanwhile, since two tethering points are required to make a single-loop brush, the grafting density of effective tethering points is 0.7 and 1.06 tethering points per nm^2^, respectively. Compared with other preparation methods reported in the literatures^49^,^60^, the grafting density of PLBs obtained herein has been greatly improved.

The reported ‘self-assembly-assisted-grafting-to' method is a fundamentally new approach to synthesize polymer brushes. The key of this new strategy is pre-assembling polymer chains into a 2D sheet. This process leads to the guided assembly of the end-functional groups of the polymer chain into a 2D pattern with a controlled areal density. Subsequent coupling the pre-assembled sheet therefore leads to well-defined polymer brushes. Advantages of this method include the following: (1) grafting densities of the polymer brushes can be easily controlled by PSC chain folding; (2) unprecedented high grafting densities of 2.12 chains per nm^2^ can be achieved; (3) PLBs can be easily synthesized and the PLBs also have well-defined structure and controlled grafting densities; and (4) the tethering points of the polymer brushes are controlled by chain folding in the PSCs, and therefore their locations on the substrates are also precisely controlled. For example, in the case of PLB, if we assume the chain-folding direction in PCL is typically (200) or (110), three-fold conformation dictates the chain end-to-end distance to be 1.12 and 1.24 nm. Similarly, five-fold conformation leads to the chain end-to-end distance of 1.87 and 2.07 nm. The tethering point of the PLB can therefore be precisely controlled. Note that since the pre-assembly process is required for the polymer brush synthesis in this study, it is limited to crystalline polymers. Nevertheless, we envisage that block copolymers containing one crystalline segment can be used for single-crystal growth to synthesize noncrystalline polymer brushes. More detailed work will be reported in the near future.

## Discussion

In this work, we report a fundamentally new ‘self-assembly-assisted-grafting-to' method to synthesize polymer brushes. End-functionalized PCL has been pre-assembled into 2D single-crystal sheets, chemical coupling of which with solid substrates led to polymer brushes. For a given molar mass, by changing the crystallization temperature, the chain-folding structures can be precisely controlled, which eventually can be used for tuning grafting density of the subsequent polymer brushes. Polymer brushes with a grafting density as high as 2.12 chains per nm^2^ have been achieved. Moreover, we have demonstrated that by using oddly folded PSCs with bi-functionalized chain end, PLBs with controlled tethering points, and grafting densities can be synthesized. Our method therefore provides a new pathway to synthesizing polymer brushes, particularly super-dense polymer brushes, with controlled architecture and grafting density.

## Methods

### Materials

(3-isocyanatopropyl)triethoxysilane (95%), dibutyltin dilaurate (95%), tin(II) 2-ethylhexanoate (92.5–100.0%) and dichloromethane (anhydrous, 99.8%) were purchased from Aldrich and used as received. Tetraethylene glycol monomethyl ether (98%) was bought from Alfa Aesar and used as received. ɛ-Caprolactone (97%) was purchased from Aldrich and distilled under reduced pressure before use. 1-butanol (⩾99.5%) was purchased from Aldrich and distilled before use. All other reagents were purchased from Aldrich and used as received.

Synthesis of model polymers, procedure of polymer single-crystal growth and brush synthesis can be found in Supplementary Methods.

### Characterizations

Proton nuclear magnetic resonance spectra of synthesized PCL were recorded on a Varian 500 MHz spectrometer using deuterated chloroform (CDCl_3_) as the solvent and tetramethylsilane as the internal standard. GPC characterizations were conducted using Waters GPC system with a 1525 binary HPLC pump, and a Waters 2414 refractive index detector was used. All measurements were performed using tetrahydrofuran as the carrier solvent with a flow rate of 1.0 ml min^−1^ at 30 °C. Standard monodispersed PS (Shodex standard, Kawasaki, Japan) were used for calibration. DSC experiments were carried out using a Perkin-Elmer DSC 7. The samples with an average weight of 2 mg were heated from 0 to 100 °C at a scanning rate of 10 °C min^−1^ under a nitrogen atmosphere and were cooled and reheated using the same rate. Phase-contrast optical microscopy images were collected using an Olympus BX-51 equipped with an Insight digital camera. Tapping-mode-AFM experiments were conducted on a Bruker Multimode 8 AFM and a Bruker Dimension Icon AFM (Bruker Nano, Santa Barbara, CA). Specifically, TESPA silicon probes (Bruker, Camarillo, CA) with spring constant *k*∼42 N m^−1^ and resonance frequency ∼320 kHz were used, and the images were acquired with 512 × 512 points at a scan rate of ∼1.0 Hz per line. Nanoscope Analysis software (Version 1.40, Bruker, Camarillo, CA) was used for image analysis to obtain polymer brush thickness and surface roughness data.

## 

## Additional information

**How to cite this article:** Zhou, T. *et al*. Towards controlled polymer brushes via a self-assembly-assisted-grafting-to approach. *Nat. Commun.* 7:11119 doi: 10.1038/ncomms11119 (2016).

## Supplementary Material

Supplementary Information Supplementary Figures 1-4, Supplementary Table 1 and Supplementary Methods

## Figures and Tables

**Table 1:** Summary of the polymer brush synthesis using mono-functionalized PCL single-crystal templates prepared at different crystallization temperatures (*T*_c_).

**Polymer**	***T***_**c**_ **(°C)**	***h***_crystal_* **(nm)**	***h***_**brush**_† **(nm)**	***R***_**q**_‡ **(nm)**	***h***_**brush**_§ **(nm)**	***R***_**q**_|| **(nm)**	***σ***_**theory**_¶ **(nm**^**−2**^**)**	***σ***_**real**_# **(nm**^**−2**^**)**
TEG-PCL30-SiOR	5	5.5	2.8	0.36	2.2	0.18	0.53	0.44
	15	9.0	3.9	0.38	3.2	0.29	0.89	0.65
	35	12.0	5.4	0.34	4.6	0.23	1.33	0.94
TEG-PCL16-SiOR	30	14.0	6.6	0.42	5.6	0.28	2.67	2.12

^*^Thickness of single-crystal lamellae measured using AFM.

^†^As-prepared polymer brush thickness measured using AFM.

^‡^Root mean squared (r.m.s.) average of height deviations of polymer brush-covered regime obtained from AFM images of as-prepared polymer brushes.

^§^Polymer brush thickness after thermal annealing obtained from AFM.

^||^R.m.s. average of height deviations of polymer brushes covered regime obtained from AFM images of polymer brushes after thermal annealing.

^¶^Theoretical grafting density of polymer brushes assuming all alkoxysilane chain ends on one side of the crystal are coupled with the glass surface.

^#^Calculated grafting density of polymer brushes using equation (1) based on experimental results.
