# Supplementary material for: Towards controlled polymer brushes via a self-assembly-assisted-grafting-to approach
Source: Nat Commun. 2016 Mar 24;7:11119. doi: 10.1038/ncomms11119 (PMC4820851; doi:10.1038/ncomms11119)
Supplement: Supplementary Information — Supplementary Figures 1-4, Supplementary Table 1 and Supplementary Methods [file ncomms11119-s1.pdf]

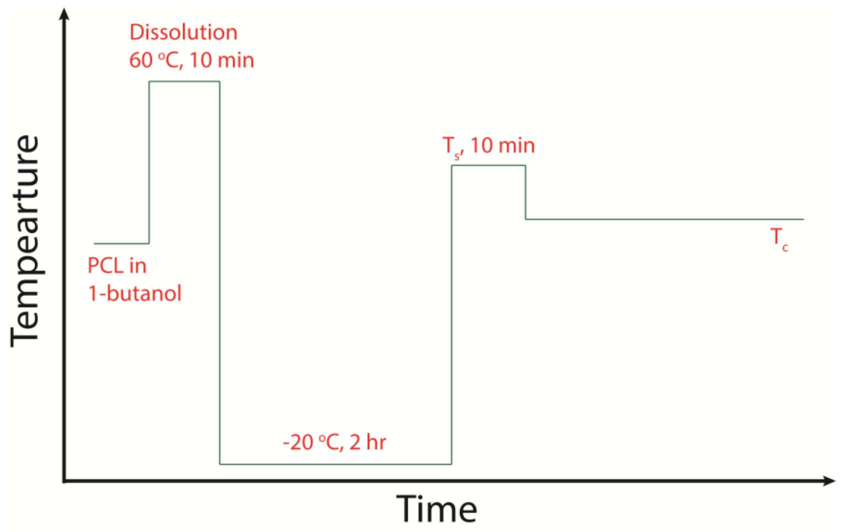

Supplementary Figure 1. Temperature profile of self-seeding method for polymer single crystal preparation in dilute solution.

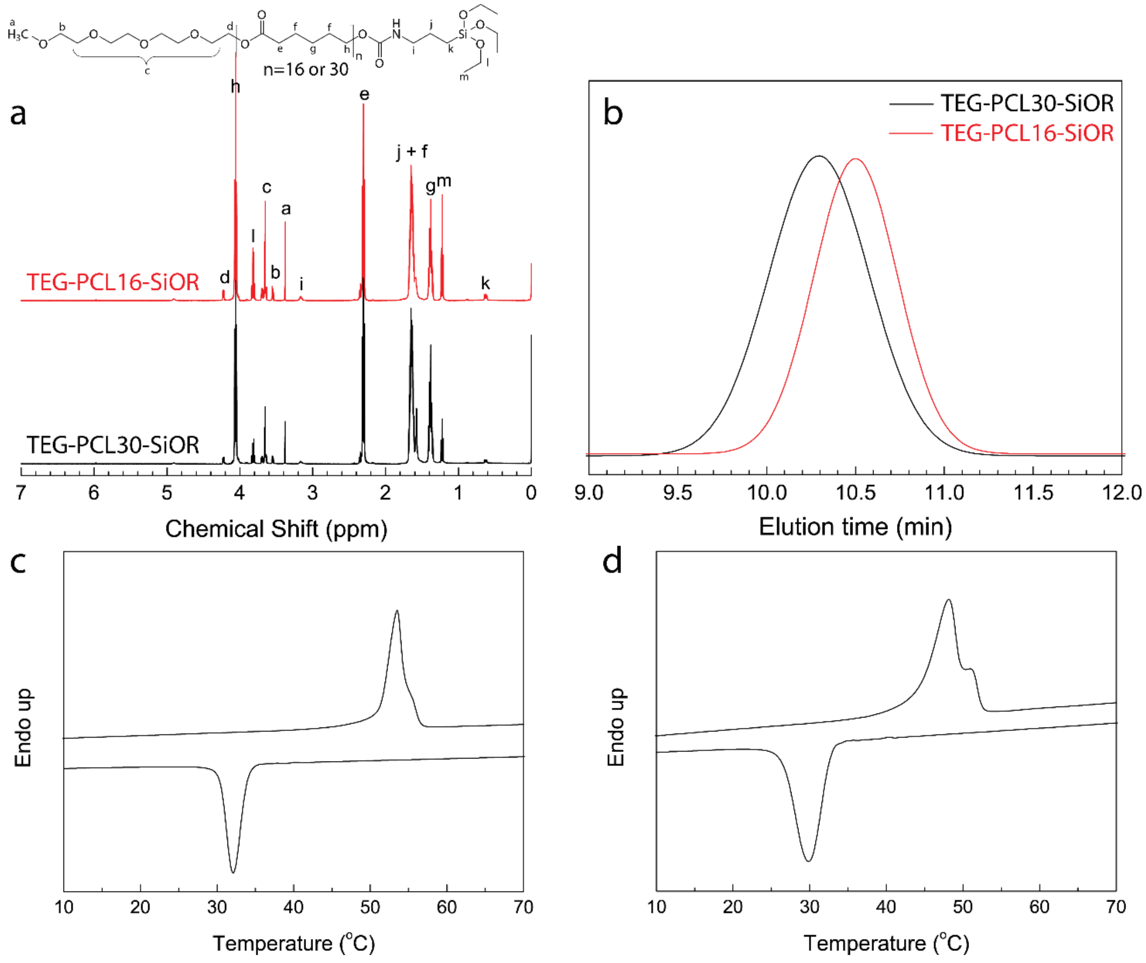

Supplementary Figure 2. ^{1}H nuclear magnetic resonance (NMR) spectra (a) and gel permeation chromatography (GPC) curves (b) of TEG-PCL30-SiOR and TEG-PCL16-SiOR; (c) Differential scanning calorimetry (DSC) first cooling and second heating curves of TEG-PCL30-SiOR; (d) DSC first cooling and second heating curves of TEG-PCL16-SiOR.

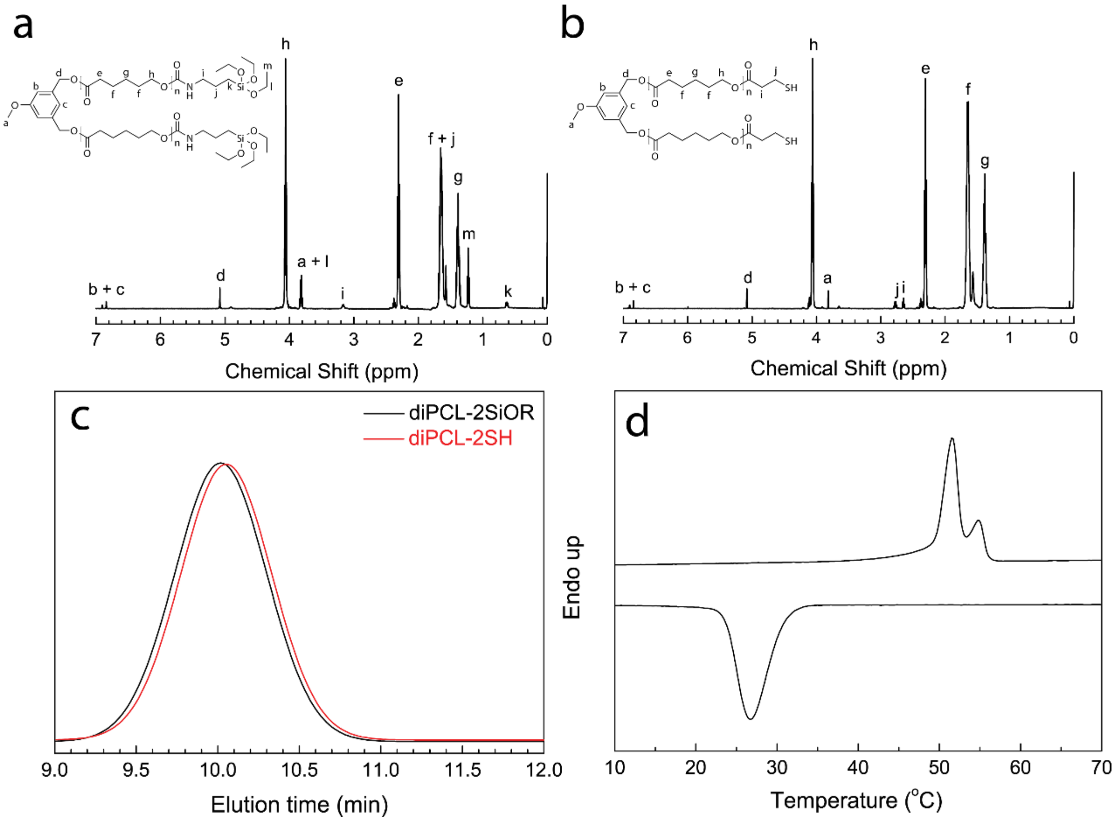

Supplementary Figure 3. ^{1}H nuclear magnetic resonance (NMR) spectra (a & b) and gel permeation chromatography (GPC) curves (c) of diPCL-2SiOR and diPCL-2SH; (d) Differential scanning calorimetry (DSC) first cooling and second heating curves of diPCL-2SiOR.

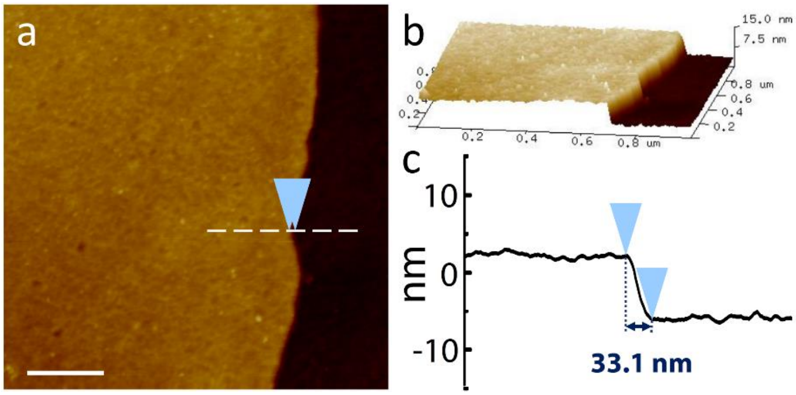

Supplementary Figure 4. (a) Tapping-mode atomic force microscopy (TM-AFM) height image of the interface between two overlapping single crystals prepared from TEG-PCL30-SiOR grown at 15 ^{o}C; (b) 3D height image of (a); (c) Height profile of the corresponding section line in (a). The width of the overlapping interface is 33.1 nm based on the step width (average of 10 independent measurements). The scale bar in (a) is 200 nm.

Supplementary Table 1. Summary of the combination of seeding temperature (T_s) and crystallization temperature (T_c) for individual PCL sample used in this study.

Polymer	T_s (^oC)	T_c (^oC)
TEG-PCL30-SiOR	42	5
		15
		35
TEG-PCL16-SiOR	39	30
diPCL-2SiOR	41.5	5
		35
diPCL-2SH	43	35

Supplementary Methods

Synthesis of PCL with single alkoxysilane terminal group (TEG-PCL30-SiOR & TEG-PCL16-SiOR)

Two PCL samples with hydroxyl terminal groups and different degree of polymerization (30 and 16, denoted as TEG-PCL30-OH and TEG-PCL16-OH, respectively) were firstly synthesized using tetraethylene glycol methyl ether as initiator. For the synthesis of TEG-PCL30-OH, 1.545 g CL, 0.2 g initiator and 31 μL tin(II) 2-ethylhexanoate were mixed in a Schlenk flask until homogeneous. The mixture was degassed and refilled with nitrogen 5 times before it was immersed into an oil bath at 110 oC. After 24 hr, the flask was quenched to room temperature to stop reaction. The mixture was then dissolved in dichloromethane and added dropwisely into cold methanol toG-OH, everything else was kept constant while 0.42 g initiator was added instead of 0.2 g.

Alkoxysilane functionalization of TEG-PCL30-OH and TEG-PCL16-OH was conducted using (3-isocyanatopropyl)triethoxysilane with dibutyltin dilaurate as catalyst. In a typical reaction, 150 mg hydroxyl-teminated PCL was dissolved in 10 mL anhydrous dichloromethane. To this solution 0.7 mL (3-isocyanatopropyl)triethoxysilane and 0.85 μL dibutyltin dilaurate were added. The reaction proceeded under the protection of nitrogen for 12 hr before the functionalized PCL was collected by precipitating the solution into cold hexane. The functionalized polymer (TEG-PCL30-SiOR and TEG-PCL16-SiOR) were stored under vacuum at room temperature for 24 hr before use.

Synthesis of α, ω-functionalized PCL (diPCL-2SiOR & diPCL-2SH)

First of all, α, ω-hydroxyl terminated PCL (diPCL-2OH) was synthesized using (5-methoxy-1,3-phenylene)dimethanol as the initiator. To a Schlenk flask 1.545 g of CL (13.5 mmol), 56.8 mg of (5-methoxy-1,3-phenylene)dimethanol (0.34 mmol) and 14 mg of tin(II) 2-ethylhexanoate (0.034 mmol) were added. The flask was degassed and refilled with N_2 5 times before brought to 110 °C for polymerization. The reaction was stopped after 24 hr and the solid was dissolved in dichloromethane and precipitated in cold methanol. The polymer appeared as white powder was collected by filtration and stored under vacuum at room temperature for future reaction.

For the synthesis of diPCL-2SiOR, 100 mg of diPCL-2OH was dissolved in 10 mL anhydrous dichloromethane. To this solution 0.5 mL of 3-(triethoxysilyl)propyl isocyanate and 0.6 mg of dibutyltin dilaurate was added. The reaction was allowed to proceed at room temperature for 12 hr. After the reaction was stopped, the solution was concentrated using rotary evaporator and precipitated into cold methanol. DiPCL-2SiOR was collected via filtration and stored under vacuum at room temperature for characterizations and further reaction.

For the synthesis of diPCL-2SH, 100 mg of diPCL-2OH was dissolved in 3 mL 3-mercaptopropionic acid. The solution was degassed and refilled with N_2 three times, and 20 mg of CALB was added into the solution, followed by two additional degas-refill N_2 cycles. The reaction was carried out at 60 ^oC for 48 hr before the solution was precipitated into cold methanol. DiPCL-2SH was collected by filtration and stored under vacuum at room temperature for characterizations and further reactions.

Crystallization of functionalized PCL with controlled lamellae thickness via self-seeding method

For all PCL samples, self-seeding method was used to grow single crystals in dilute 1-butanol solution with concentration around 0.03 wt.%. In a typical crystallization process, PCL was firstly dissolved in 1-butanol at 60 ^{o}C. The solution was then stored at -20 ^{o}C for 2 hr to allow fast crystallization of PCL. Immediately after that, the crystal suspension was kept at seeding temperature (T_s=42 ^{o}C) for 10 min to form crystal seeds. After that, PCL was allowed to crystallize at specific T_c for at least 48 hr before the single crystals were separated from the solution via multiple centrifugation. The crystals were finally redispersed in 1-butanol for further reactions. Different seeding temperature (T_s) and crystallization temperature (T_c) were chosen for different PCL samples as summarized in **Supplementary Table 1**.

Preparation of PCL brushes on solid substrates using PSCs as templates

Single-tethered polymer brushes (STPBs) on flat glass surfaces were prepared using PSCs grown from TEG-PCL-SiOR as templates. Polymer loop brushes (PLBs) were prepared using PSCs grown from diPCL-SiOR as templates. Before all immobilization process, the glass substrates were cleaned using hot piranha solution for 30 mins to remove organic contaminants. PSCs were firstly deposited onto the substrates via spincoating method in order to form separated islands of PSCs for detailed atomic force microscopy (AFM) characterizations. After the deposition process, the coated glass slides were transferred to a sealed chamber to which 300 μL of ammonia was added. The reaction was allowed to proceed for 20 min before the ammonia was removed, and the coated glass slides were washed thoroughly using acetone by sonication bath for 10 min.

After that, the substrates were blown dry using nitrogen at ambient condition, and stored at room temperature for characterizations.

To prepare polymer brush coating with macroscopic coverage on glass substrate, dropcasting of PSCs instead of spincoating was adopted. After the solvent was completely evaporated, the polymer chains were allowed to reaction with glass surface via similar ammonia-catalyzed procedure described above. The coated substrate was also washed thoroughly using acetone in sonication bath for 10 min before further characterizations.

PCL loop brushes on flat gold substrate were prepared using PSCs grown from diPCL-2SH as templates. The Au substrates were prepared by thermal evaporation deposition of Cr followed by Au on a clean Si wafer, and cleaned using hot piranha solution prior to crystal immobilization. To prepare brushes, diPCL-2SH PSCs were dropcasted from crystal suspension in 1-butanol onto Au substrate. After the solvent evaporated, the PSCs were allowed to reaction with Au under vacuum for 2 days. The unbounded polymer was then washed away by acetone via sonication for 10 min and dried under nitrogen. The coated substrates were stored under vacuum until further characterizations. To oxidize either diPCL-2SH PSCs or PLBs on Au substrates, the coated substrates were left under ambient lab condition for 2 days. After that, these functionalized surfaces were used for X-ray photoelectron spectroscopy (XPS) measurements.
